# Primary Follicular Lymphoma of the Duodenum with Erosions as Atypical Macroscopic Features

**DOI:** 10.1155/2012/582607

**Published:** 2012-05-29

**Authors:** Keiko Takeuchi, Masaya Iwamuro, Atsushi Imagawa, Yoshitsugu Kubota, Katsuya Miyatani, Katsuyoshi Takata, Hiroyuki Okada

**Affiliations:** ^1^Department of Gastroenterology, Mitoyo General Hospital, Kan'onji 769-1695, Japan; ^2^Department of Gastroenterology and Hepatology, Okayama University Graduate School of Medicine, Dentistry, and Pharmaceutical Sciences, Okayama 700-8558, Japan; ^3^Department of Transfusion Medicine, Faculty of Medicine, Kagawa University, Kita-Gun 761-0793, Japan; ^4^Department of Pathology, Mitoyo General Hospital, Kan'onji 769-1695, Japan; ^5^Department of Pathology, Okayama University Graduate School of Medicine, Dentistry, and Pharmaceutical Sciences, Okayama 700-8558, Japan; ^6^Department of Endoscopy, Okayama University Hospital, Okayama 700-8558, Japan

## Abstract

A 52-year-old Japanese woman who was eventually diagnosed with primary follicular lymphoma of the duodenum showed atypical endoscopic features, namely, erosions with peripheral whitish edematous mucosa. Initial biopsy specimens taken from the erosions revealed insufficient numbers of lymphoma cells for histological diagnosis. Subsequent biopsy specimens from the peripheral mucosa containing the whitish enlarged villi showed infiltration of the lymphoma cells forming lymphoid follicles, which led us to the appropriate diagnosis. This case indicates that endoscopists should take biopsy samples from the peripheral mucosa with whitish enlarged villi rather than erosions in the rare instances that erosions appear as the main macroscopic feature of intestinal follicular lymphoma.

## 1. Introduction

 Primary intestinal follicular lymphoma is a variant of nodal follicular lymphoma, and the majority of cases involve the duodenum [1–3]. The presence of small whitish polypoid nodules up to 2 mm in diameter is the key endoscopic feature of the disease, which has been described in various terms such as multiple polypoid lesions, multiple small polyps, multiple nodules, or multiple granules [4, 5]. Erosions or ulcers are relatively infrequent macroscopic features in duodenal follicular lymphomas.

 Herein, we present a case of primary duodenal follicular lymphoma with erosions as the main endoscopic feature. The peripheral mucosa of the erosions was edematous and whitish but lacked the polyps or nodules that are typical of the duodenal follicular lymphomas. It is noteworthy that biopsy samples taken from the erosions at the first esophagogastroduodenoscopy contained insufficient lymphoid cells to make a definitive diagnosis. Subsequent biopsy samples from the whitish mucosa around the erosions included small-to-medium-sized lymphoid cells that formed lymphoid follicles and infiltrated the duodenal villi. These features and the immunohistochemical findings led us to the appropriate diagnosis of follicular lymphoma. The endoscopic and pathological features of the presented case are discussed.

## 2. Case Presentation

A 52-year-old Japanese woman presented to the Mitoyo General Hospital with intermittent epigastralgia lasting for one week. She had been taking antipsychotic agents, namely, risperidone, and milnacipran hydrochloride, for treatment of schizophrenia. She had a past history of eradication of *Helicobacter pylori* 10 years previously, and thereafter she had undergone annual followup by routine esophagogastroduodenoscopy. Esophagogastroduodenoscopy performed two years ago demonstrated no abnormality except for atrophic gastritis, and neither *Helicobacter pylori* infection nor lymphomatous lesions, including extranodal marginal zone B-cell lymphoma of mucosa-associated lymphoid tissue, were detected in the biopsy samples from the stomach. Esophagogastroduodenoscopy performed one year ago also showed only atrophic gastritis. Physical examination revealed no abnormalities, and there was no evidence of hepatosplenomegaly or peripheral lymphadenopathy. All laboratory findings, including the levels of lactate dehydrogenase (LDH) and soluble interleukin-2 receptor (sIL-2R), were within the normal ranges.

**Figure 1:**
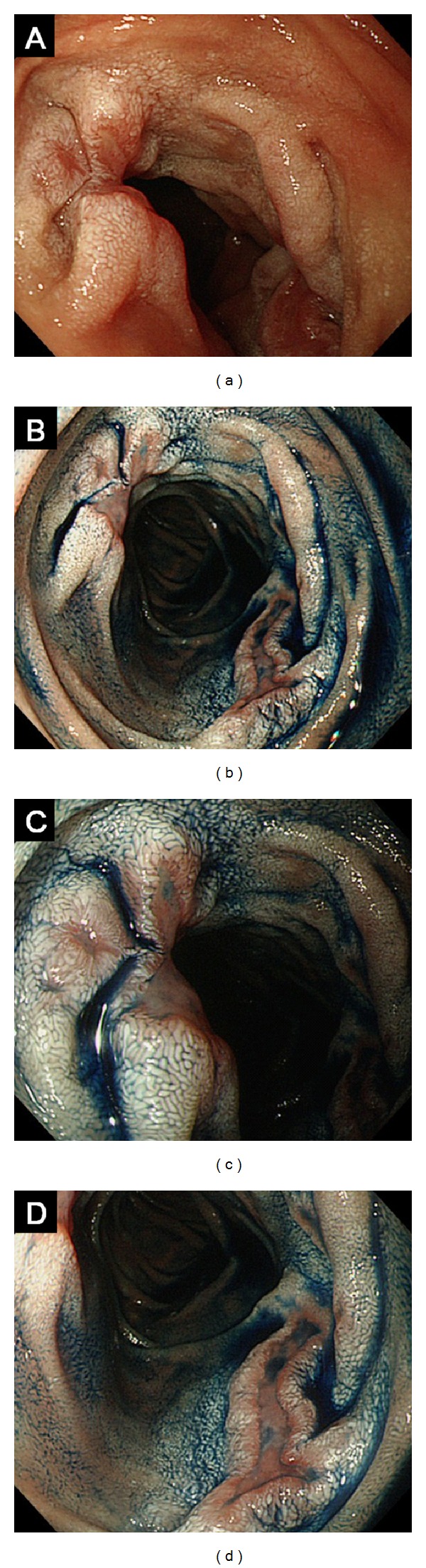
Endoscopic images of the duodenal second portion. Irregular erosions with peripheral edematous whitish mucosa were seen (a). The erosions were emphasized by indigo carmine dye spraying (b). Close-up views of the lesions demonstrated complete loss of villi within the erosions and whitish enlarged villi in the peripheral mucosa (c and d).

**Figure 2:**
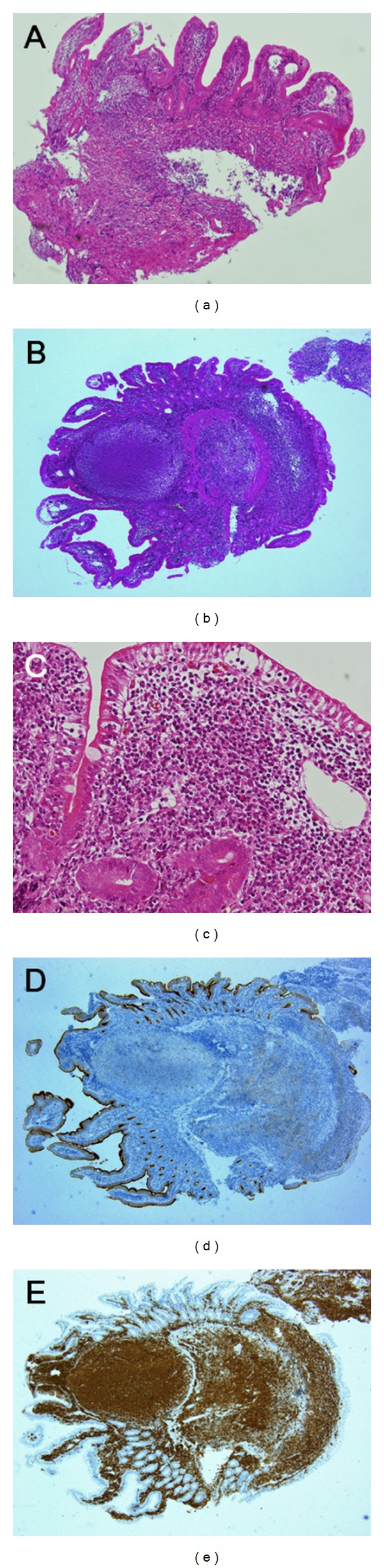
Histological findings of the biopsy samples. The initial biopsy specimens taken from the border of erosions contained small number of duodenal villi. Lymphoma cells were scanty, and therefore, the specimens were inadequate for pathological diagnosis ((a) hematoxylin and eosin staining, original magnification ×40). Second biopsy specimens taken from the peripheral whitish mucosa showed lymphoid follicles composed of small-to-medium-sized lymphoid cells ((b) hematoxylin and eosin staining, original magnification ×40). Lymphoma cells also infiltrated into the duodenal villi ((c) hematoxylin and eosin staining, original magnification ×200). Lymphoma cells were positive for CD10 ((d) original magnification ×40) and BCL2 staining ((e) original magnification ×40).

**Figure 3:**
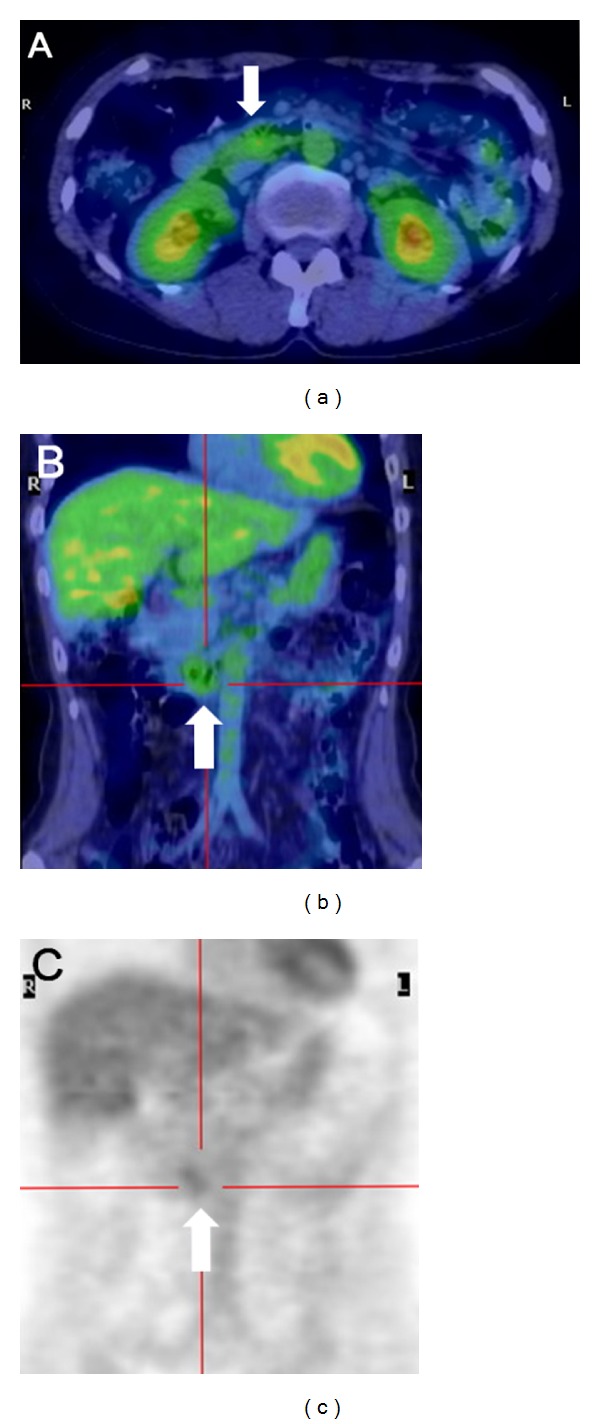
18F-FDG PET images of the patient. Positive tracer uptake in the duodenum was shown in the horizontal section ((a), arrow) and coronal sections ((b, c), arrows). No abnormal uptake in other organs including the lymph nodes was detected.

Esophagogastroduodenoscopy revealed irregular-shaped erosions aboral to the ampulla of Vater (Figure 1). The mucosa around the erosions appeared edematous and was composed of whitish enlarged villi. The lesions were soft and thus were easily deformed by deaeration. Two biopsy samples were taken from the erosions, and one sample was taken from the border of the erosions. These samples contained small-to-medium-sized lymphoid cells, but the number of lymphoid cells was not enough to make a definitive diagnosis of any type of lymphoma (Figure 2(a)). Esophagogastroduodenoscopy was then repeated, and biopsy specimens were taken from the whitish villi around the duodenal erosions. The specimen contained lymphoid follicles in the duodenal mucosa, and these were comprised of small-to-medium-sized lymphoid cells, which had also infiltrated into the villi (Figures 2(b) and 2(c)). The lymphoid cells were positive for CD20, CD10 (Figure 2(d)), and BCL2 (Figure 2(e)), but negative for CD3. Colonoscopy revealed no abnormality. Evaluation of small bowel involvement by double-balloon enteroscopy or video capsule endoscopy was proposed but was not performed as the patient refused. Computed tomography (CT) scanning of the neck, chest, abdomen, and pelvis showed neither lymphadenopathy nor a thickened gastrointestinal wall including the duodenum. 18F-fluorodeoxyglucose (FDG) positron emission tomography (PET) scanning detected positive tracer uptake in the inferior duodenal angle, whereas in other organs there were no abnormal accumulations of 18F-FDG (Figure 3). Consequently, the patient was diagnosed with primary follicular lymphoma of the duodenum. The clinical stage was considered as stage I, based on the Lugano staging system for the classification of the gastrointestinal tract lymphoma [6, 7] (stage I: confined to the gastrointestinal tract, stage II-1: extending into the abdomen from primary gastrointestinal site with local nodal involvement, stage II-2: extending into the abdomen from primary gastrointestinal site with distant nodal involvement, stage IV: disseminated extranodal involvement or concomitant supradiaphragmatic nodal involvement).

**Figure 4:**
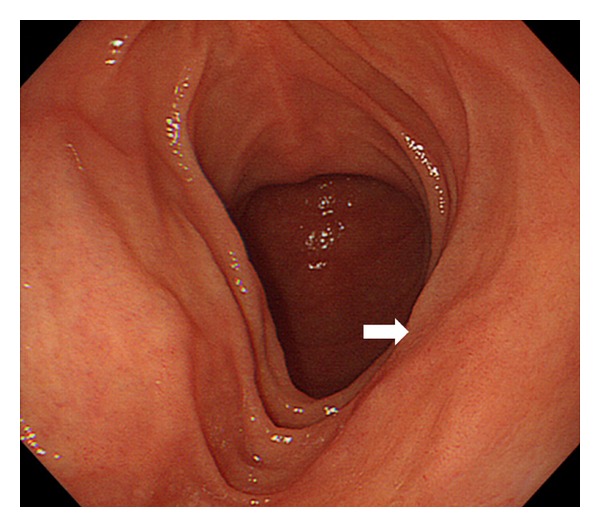
Endoscopic image after local radiation therapy. Both of the duodenal erosions and the whitish mucosa totally disappeared after completion of the radiation therapy. Only a slight deformity of the duodenal mucosa was noted, and this seemed to be a vestige of the lymphoma lesion (arrow). Histological examination confirmed complete remission of the follicular lymphoma.

 Radiation (40 Gy in 20 fractions) was conducted as a curative therapy for the patient, and the treatment was completed without any adverse events. Esophagogastroduodenoscopy was performed one month after completion of the radiation therapy, and it revealed disappearance of the lymphomatous lesions in the duodenum (Figure 4). Histological evaluation of the biopsied specimens from the duodenal mucosa also confirmed complete remission. The patient remained healthy, with no recurrence of lymphoma for 20 months after the radiation therapy.

## 3. Discussion

**Figure 5:**
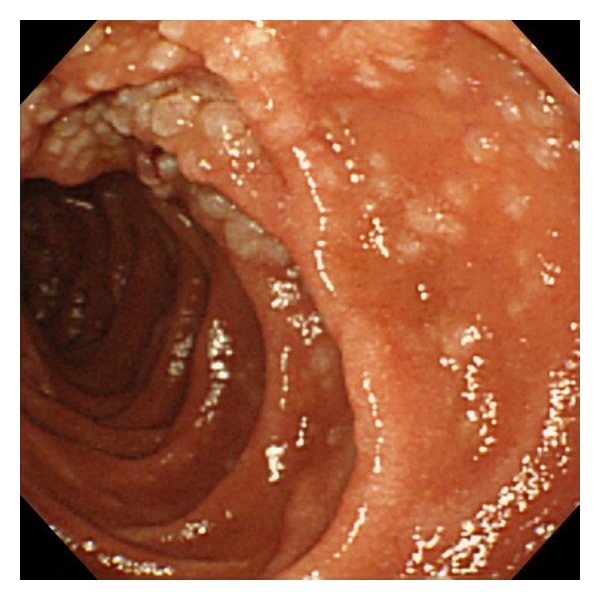
Typical endoscopic image of follicular lymphoma of the duodenum observed in another patient (a 60-year-old man). Small whitish polypoid nodules were seen. In this patient, the entire gastrointestinal tract was evaluated by esophagogastroduodenoscopy, colonoscopy, and video capsule endoscopy. No involvement of other gastrointestinal tract was detected.

 Yamamoto et al. summarized the cases with gastrointestinal follicular lymphoma reported in the English-language literature, and they noted that, among 133 cases with duodenal follicular lymphoma, multiple nodular lesions were found in 119 (89%) cases, mass lesions with or without ulcer in 5 (4%), polyps in 6 (5%), and other macroscopic features in 3 (2%) [5]. Recently, Takata et al. have reported 125 Japanese patients with primary gastrointestinal follicular lymphoma [2]. In their report, the macroscopic features of 110 patients were subcategorized as multiple nodules in 88 (80%), polyps in 13 (12%), ulcers in 5 (5%), diffuse disease in 2 (2%), and unclassified findings in 2 (2%). Therefore, multiple nodules were the predominant endoscopic feature (Figure 5), accounting for 80–89% of patients with gastrointestinal follicular lymphoma, and erosions as described in the presented case are considered to be rare.

 Erosions in the duodenum are frequently seen as nonspecific duodenitis or in peptic ulcer disease due to *Helicobacter pylori* infection or drugs (e.g., nonsteroidal anti-inflammatory drugs). The differential diagnosis of erosions include other relatively infrequent etiologies, such as cytomegalovirus infection [8], vasculitis [9], amyloidosis [10], gastrinoma, and duodenal adenocarcinoma. On the other hand, whitish lesions in the duodenum may be caused by various etiologies, such as normal lymph follicles, intestinal lymphangiectasia, chronic non-specific duodenitis, and giardiasis [11]. In our present case, biopsy was performed for pathological diagnosis because the duodenal lesions seemed to be different from the common non-specific duodenitis or peptic ulcer disease.

 Initial biopsy samples were taken from the erosions where the villi and part of the mucosal layer had disappeared. Those samples were insufficient for histological diagnosis owing to scanty lymphoma cells. In contrast, biopsy samples taken from the peripheral mucosa with whitish enlarged villi contained a greater number of lymphoma cells forming lymphoid follicles. Generally, neoplastic lymphoid follicles composed of lymphoma cells are confined to the mucosal layer and/or submucosal layer of the gastrointestinal tract [5, 12–14]. Moreover, whitish enlarged villi are reportedly a characteristic feature of intestinal follicular lymphomas in magnifying endoscopy [13, 15–18]. We speculate that these whitish enlarged villi reflect the infiltration of lymphoma cells within the villi. Consequently, for the histological diagnosis of gastrointestinal follicular lymphomas, biopsy samples should be taken not only from the erosions or ulcers but also from the whitish mucosa with enlarged villi, where lymphoma cells may be mainly present. Accordingly, as a common practice, biopsies from the margin of erosions or ulcers have to be done to securely exclude adenocarcinoma. In this paper, we emphasize the importance of additional biopsy from the peripheral mucosa when endoscopists observe whitish enlarged villi around the erosions or ulcers.

No strategy for staging of gastrointestinal follicular lymphoma has been established yet. For staging of follicular lymphomas of nodal origin, the European Society of Medical Oncology (ESMO) clinical practice guidelines do not recommend routine use of PET scanning, but these guidelines suggest that PET scanning has a valuable role to confirm the disease stage as “limited” (stage I or II) and to avoid futile radiotherapy in patients with widespread disease [19, 20]. Several studies support the significance of 18F-FDG-PET for initial staging of nodal follicular lymphoma [20–22]. In contrast, the sensitivity of PET scanning for detecting gastrointestinal lesions of follicular lymphoma is lower than that for nodal lesions [5]. Hoffmann et al. described eight patients of duodenal follicular lymphoma who showed negative tracer uptake in the duodenal lesions [23]. However, Takada and Yamamoto reported a patient with gastric follicular lymphoma and positive tracer uptake in the extragastrointestinal lesion, which was not visualized by CT scanning [24]. They suggested the potential utility of PET scanning for detection of the extra-gastric involvement. Therefore, in our present case, we performed PET scanning as a complementary method for staging, in addition to CT scanning of the neck, chest, abdomen, and pelvis.

 In conclusion, we presented herein a patient with primary follicular lymphoma of the duodenum showing erosions as atypical macroscopic feature. We suggest that endoscopic biopsy should be taken from the peripheral mucosa containing whitish enlarged villi, rather than the erosions themselves.
